# Hygiène

**Published:** 1836-10

**Authors:** 


### HYGIÈNE.

### *On the Causes of Colica Pictonum amongst the Workmen who prepare White Lead.* By A. Chevallier.

[In a previous number we gave the results of M. Chevallier's careful researches into the diseases to which printers are subject; he has now, with similar industry and skill, investigated the effects of white lead on workmen, with a view to the amelioration of their condition.]

Workmen who use lead in any way are liable to contract "the painter's colic;" bût M. Chevallier has confined himself to the study of this disease among those employed in manufacturing white lead *(ceruse)* only. For this purpose the following questions were addressed to the manufacturers, chemists, and medical men attached to the manufactories or to the hospitals to which the patients were sent, and the answers contain an analysis of their replies.

1. Are the manufacturers of white lead numerous in France ?—About 450.

2. Are women and children employed as well as men ?—Women, and children from fourteen to fifteen, are employed.

3. Are the men, the women, or the children most subject to colic ?—Some manufacturers say the men; but others, who employ women, affirm that they are most liable from their susceptibility. Dr. Renauldin thinks that, if women are less liable, it is because they are not employed in powdering the white lead. It has been remarked that those of a nervous constitution are most liable to it, and the disease is more severe. Tables kept by physicians attached to manufactories are required to solve this question.

4. Does the disease affect all the workmen, or one workman many times ?

The answers differ, but they prove: 1. That many workmen pass whole years without an attack; but these are men born in the country, who take much milk, and systematically avoid all excesses. 2. Relapses are frequent, and particularly so if the workman goes back to his work before he has regained his strength. 3. Imprudence and excess, rather than the labour itself, predispose to the disease. 4. Some men work for three years without disease, others are attacked in two or three weeks. Dr. Renauldin says, all are destined to have the disease: M. T. Desplanches says, all have it, because precautions are not taken in the establishments.

5. Does the disease attack the same workmen many times ?—Yes; and relapses are most frequent after a violent attack. Those that have had it are most disposed to have it again, and after a second or third seizure should give up the employment. Workmen, however, have been known to have had it five, six, seven, or eight times. M. Dehéque knew an old man who had had it eleven times: he was a clever workman, but lazy, drunken, and in a constant state of brutishness; he trembled, and had lost his beard, eyebrows, and almost all his hair; he seemed always asleep, spoke with difficulty, but worked and lived hard, sleeping well on straw, eating little, drinking much in small quantities; his face was swollen, and his complexion white, soft, and shining.

6th. Is the temperature a predisposing cause of the disease ?—At Lille it was remarked that the disease was more violent during intense heat and great cold; and this is confirmed by the admissions into La Charité in 1833; for, during January there were fifteen individuals admitted with this colic, and in July forty-five. At Strasbourg the disease was most frequent in damp winters. M. Fee says that the workmen have observed that those who live in the manufactory are more liable to it than the others; and that those who were obliged to walk some distance into the country to their homes were hardly ever seized.

7th. Are these diseases ever serious or fatal ?—1. When suitable precautions are taken the disease is not serious, particularly if the workmen cease to work at the white-lead. 2. It may be more serious if there are complications. 3. After a third or fourth attack, it may produce acute pains in the limbs, and in the end paralysis and incapacity to work. 4. The first or second attacks, when well treated, leave no dangerous sequelæ. 5. Death may ensue if there is negligence on the part of the workman. Dr. Renauldin says on this subject, that "workmen who have had it as many as six times often die, particularly if it reacts on the brain, producing epilepsy, which is rapidly fatal notwithstanding every attention, or the limbs become paralytic; which state is very difficult to cure, and often incurable." Out of 3,569 cases of painter's colic reported by various authors, there are ninety-five deaths, or a mortality of little less than one in thirty; and this is owing to complications or affections of the nervous system.

8th. How long may a workman work in a white-lead establishment?—Those who are regular in their conduct, sober, and use much milk, may work for an indefinite period. Among eighty-two workmen in M. T. Lefebvre's establishment, a great part have worked three, four, five, six, and seven years without suffering from such attacks as incapacitated them. In other places similar observations cannot be made, as the men only work there when there is no employment elsewhere. "There are a number of instances of men working twenty or thirty years where the ceruse is made by the Dutch process." (M. Stollé, of Strasbourg.)

9th. Do the workmen die young?—"Those who are most susceptible of it always die prematurely, and those who have the most power of resistance die before their natural term." (Dr. Renauldin.) "The trade does not seem to influence the length of their life, but they become old and infirm prematurely." (T. Desplanches.)

10th. Are any precautions taken?—Numerous measures have been adopted, and attempts made to combat the carelessness of the workmen, who will not willingly follow the recommendations. The men have been recommended not to touch the oxide of lead unless it is wet, to avoid the emanations from melting lead, and the dust of white leads; to cover their mouths with a wet cloth, and their heads with linen whilst packing the ceruse in barrels; to use great cleanliness, washing their hands and changing their outside garments before eating; to drink milk and beer: when this advice has been taken, the men have been rarely attacked, and spacious shops, ventilated by free currents of air, have diminished the disease.

11th. Are medical men attached to these manufactories?—To some of them only: in others, the sufferers are immediately sent to an hospital. In Germany and Holland, where ceruse manufactories are very numerous, the government obliges the manufacturers to intrust the health of their workmen to a medical man.

12th. What parts of the process appear to be particularly hurtful?—1. The separation of the layers of oxidized and carbonated lead from that which is not so. 2. The remelting of the fragments of lead. 3. Packing the ceruse in barrels. 4. Sifting the ceruse.

13th. What precautions are or could be taken to render these processes less dangerous?—In some manufactories where the fragments of lead are remelted, there is a lofty ventilating chimney, with a ventilator of sufficient power to expel instantly the noxious vapour; in others the sieves and mills are enclosed in wooden frames, forming partitions separating this part of the workshop from the rest; also, when the barrel of ceruse is moved, it is covered with a sheep's skin. The following plan has been adopted for some years by M. Reboul, of Pézénas:—

The melted lead is poured on plates of copper in thin layers, a foot long and eight inches broad, which are rolled up spirally, so as to be eighteen or twenty lines in diameter: these are placed in a vessel, and distilled vinegar poured over them: they are then withdrawn and exposed to the air: they are next placed in deal boxes, having a grating at the bottom, and seven or eight of these boxes (which are of the same size,) are piled one on the other in a drying place. The base of each pile is a reservoir of stone, or of wood lined with lead, having a waste-pipe passing through the wall. In the middle of the room is a charcoal furnace, over which is placed a copper vessel half filled with water. In the ceiling are holes closed by wooden trap-doors, corresponding in number to the piles of boxes, through which distilled vinegar or vinegar holding lead in solution, is poured in every morning: after this the trap-doors are closed, and during the day smaller quantities are frequently poured in through holes made in the trap-doors, which dropping guttatim from box to box and thence into the reservoir facilitates in its passage the formation of the carbonate of lead. The liquor which has not been absorbed runs out of the reservoir, and is used again. When the lead is almost entirely converted into ceruse, water is poured over it, instead of vinegar, to wash away any vinegar or acetate. The lead, thus changed into ceruse, is thrown into a large tub of water, and agitated with a spatula; the ceruse is thus separated from the non-carbonized lead and particles of oxide, which sink, and is held in suspension by the agitation of the water with which it flows out into other tubs, and subsides. This separates the greatest part of the ceruse: to obtain the rest, the residue is placed in a wooden barrel with some pieces of quartz; water is added, the barrel revolved, and the powders of lead allowed to fall again into the tub. Another washing separates the ceruse, and the grey powder which remains is spread over new rolls of lead which are placed in boxes for the drying stove. The other manipulations relating to grinding and drying it are common to all manufactories. The novelty in the process consists in the arrangement of the boxes and drying stove; in washing the lead with vinegar and solution of acetate, and in two ways, rapidly and slowly; and in the use of the barrel and quartz.

M. Reboul says, in a letter, dated 2d March, 1834, that he has used this plan for fifteen years; during the former part of this period many workmen were attacked by the lead colic, but for the last nine years there has been no case. This is simply owing to avoiding the vapours of the salts of lead and not respiring the air charged with the dust of these salts; two precautions which could be carried into effect in the process he adopts, as the ceruse is prepared in drying stoves, which need not be entered until they are cooled and ventilated; and the ceruse thus prepared being in the state of paste, need not be powdered, but only washed, dried, and packed.

14th. Are any preservatives against lead colic known in the manufactories?—No actual preservative is known, but precautions are taken which render accidents less frequent. In some manufactories, milk, butter, beer, purgatives, water acidulated with sulphuric acid, are given to the workmen; but the best preservative is a glass of water impregnated with sulphuretted hydrogen, taken every night on leaving the workshop: the water of Barèges or of Enghien will answer the same purpose.

15th. What are the precursory signs of the disease?—According to the manufacturers, those who are about to be attacked appear depressed, the face wrinkled, pale and yellow, the eyes hollow; they are low-spirited, and do not eat; there is a yellowish tint round the nose and mouth, the lips tremble and are cold, and there is constipation. M. Renauldin says, all the workmen are pale whether ill or not, and their teeth of greyish colour approaching that of lead; but the true precursory sign is numbness of the arms and legs, three or four days after which the colic comes on.

Such is a summary of the principal facts obtained by M. Chevalier; and he concludes by giving a series of directions to the manufacturers and workmen, with a view to diminish the disease, and adds a new mode of treatment.

*Advice to Ceruse Manufacturers.*

In order to preserve the health of the workmen, it is advisable—1st, to enforce strict cleanliness, and washing the hands before eating and leaving the shop, and to prevent eating in the workshops; 2d, to establish thorough ventilation; 3d, employ means to separate the scales of white lead with as little dust as possible. (For this purpose the proposition of M. D'Arcet, to pass the leaves of lead between a grooved cylinder enclosed in a wooden frame, instead of beating them, may be attended to.) 4th, to isolate the mills and sieves, and to place them in close wooden frames. 5th. That when there is dust of the ceruse in the workshop, the workmen should cover their mouths and noses with a handkerchief slightly moistened. 6th. To provide a medical attendant to examine the workmen frequently. 7th. To require those who have any precursory symptoms to leave off work for one or more days, according to the opinion of the medical man. 8th. To oblige the workmen to wear smock-frocks, (*blouses*), and to require them to leave these garments in the workshop, and to have them washed from time to time, and to reason with them on their carelessness, to which the majority of accidents are traced. 9th. To prevent debauchery among the workmen; to employ only sober men, and to discharge those who persist in drinking. 10th. To introduce the plan of drinking daily on leaving work a glass of water charged with sulphuretted hydrogen, to neutralize the effects of the ceruse which might have been absorbed. (This is easily made by passing into water sulphuretted hydrogen gas disengaged from sulphuret of iron by weak sulphuric acid.) 11th. To try means which may probably be preservative that are suggested by the medical man attached to the manufactory. 12th. To use the least dangerous processes.

*Advice to Workmen.*

1. To be sober and abstain from wine and venereal excess. 2. To be extremely clean. 3. To take solid nourishment, and not to eat largely on Sunday and Monday, and then scantily during the rest of the week. 4. To inform their master whenever they feel symptoms of indisposition; such as loss of appetite, or of spirits, numbness in the limbs, &c.

*New Treatment of Diseases produced by Lead.*

The liver of sulphur has been proposed since 1777 by Navier, as a counter-poison to lead; but he did not confirm its efficacy by actual experiment, and Orfila having shown its inapplicability, it fell into oblivion. In 1814, M. Chevalier, having convinced himself that sulphuret of lead had no action on dogs, whilst carbonate of lead was injurious, inferred that hydrosulphuric acid might be advantageously employed as a counterpoison to the salts of lead; and four years afterwards, being in a manufactory where two men were attacked with violent lead colic, he gave them about a pint of hydrosulphuretted water, which he found in the laboratory, with immediate relief. He subsequently found similar benefit in his own person. M. Ratier has since confirmed these facts by many trials at the Hospital of La Charité, and gives the following directions. Three indications are to be fulfilled:

1. To neutralize the poison, by giving internally a quantity of hydro-sulphuretted water, proportioned to the known or supposed quantity of the salt or oxide of lead absorbed. M. Rayer has used the "eau d'Enghein," but either of the following artificial preparations may be substituted:

(No. 1.) Take nineteen pints of water, and add one pint of water saturated with sulphuretted hydrogen, in which twelve grains of carbonate of soda had been previously dissolved.

(No. 2.) Dissolve five grains of sulphuret of potash in a pint of water.

The more recent the colic, the more marked the effect. Many obstinate attacks have yielded to this treatment only.

2. To relieve constipation where it exists. For this purpose, M. Rayer prescribes forty-eight grains of scammony and the same quantity of jalap in twelve pills; the patient to take from two to six until they operate. If the constipation continues, a lavement, containing an ounce of senna and two or three ounces of castor-oil.

3. To relieve pain and to procure sleep. For this purpose, one grain or one and a half grain of extract of opium is given at night.

By these means, M. Rayer has rapidly relieved the effects of the salts and oxides of lead, sometimes on the second day, often on the third or fourth, and rarely beyond the sixth. He has never seen a relapse.

M. Lefebvre has communicated by letter the particulars of four cases of colica pictonum in his manufactory, all of which yielded to the sulphuretted hydrogen treatment alone. Half a drachm of sulphuret of potash was dissolved in a pint of water, half of which was taken in two doses each day. Three were cured in two days, and one in one day.

From the injurious effects which white paint made by carbonate of lead has on the healths of painters, as well as occasionally on the inhabitants of recently painted houses, it has been proposed to substitute carbonate of zinc for the lead. From a report of commissioners appointed by the Academy of Architecture in Paris, it appears that the paint made from carbonate of zinc is not unwholesome, and that it preserves its brilliancy and whiteness. At present, however, it is more expensive than carbonate of lead, which would prevent its general adoption; but, as M. Chevallier suggests, it may be very useful in painting the rooms where sulphur-baths are given, or privies where sulphuretted hydrogen is evolved, which blackens white-lead paints.

[The general circulation of information tending to the prevention of complaints produced by employments to which large bodies of men are necessarily exposed, is one of our most useful and gratifying duties. Indeed, the value of precautionary measures promising security will be gratefully acknowledged by those who have at all reflected on the task they may have frequently performed of urging a man who has a family dependent on him to leave an occupation which is prejudicial to his health, but for which alone he is properly qualified, from having spent his whole life in its acquisition, whilst, at the time they give this advice, they are conscious that the secession of one will only substitute another equally liable to suffer.]

*Annales d'Hygiène publique. Janvier,* 1836. No. 29.

